# Enhancing Biological Control Efficacy: Insights into the Feeding Behavior and Fitness of the Omnivorous Pest *Lygus lineolaris*

**DOI:** 10.3390/insects15090665

**Published:** 2024-08-31

**Authors:** Mireia Solà Cassi, François Dumont, Caroline Provost, Eric Lucas

**Affiliations:** 1 Laboratoire de Lutte Biologique, Département Des Science Biologiques, Université du Québec à Montréal, 8888, Succursale Centre-Ville, Montreal, QC H3C3P8, Canada; lucas.eric@uqam.ca; 2 Centre de Recherche Agroalimentaire de Mirabel, 9850 Rue de Belle-Rivière, Mirabel, QC J7N 2X8, Canada; fdumon@cram-mirabel.com (F.D.); cprovost@cram-mirabel.com (C.P.)

**Keywords:** diet, integrated pest management, miridae, phytozoophagous, strawberry, tarnished plant bug

## Abstract

**Simple Summary:**

This study on *Lygus lineolaris*, an omnivorous insect pest threatening agriculture in the Neartic region, explores how different diets affect its performance in terms of survivorship, developmental time, and adult weight and length. The results highlight the significant impact of diet source, feeding regime, and the number of diet items on its performance. Observed benefits include higher survival rates, faster development, and larger and heavier adults. Feeding on strawberry results in low insect fitness, while canola-rich diets notably improve it. Furthermore, introducing multiple diet items in phytozoophagous regimes enhances overall performance. Recognizing *L. lineolaris’* nutritional needs is vital for effective pest management strategies and provides valuable insights for developing biological control programs against this agricultural threat.

**Abstract:**

*Lygus lineolaris* (Palisot de Beauvois) (Hemiptera: Miridae), a true omnivorous insect, poses a significant threat to agriculture in the Neartic region. Understanding the feeding behavior of *L. lineolaris* is crucial for developing integrated pest management strategies. This study aimed to evaluate the effects of different diets on the fitness of *L. lineolaris*, with a focus on the diet source, feeding regime (phytophagy, zoophagy, and phytozoophagy), and number of diet items. The experimental design in the laboratory investigated the impacts of strawberry, canola and buckwheat flowers, as well as spider mites and aphids to explore relationships found in a conventional strawberry field. Results reveal that diet source, feeding regime, and the number of diet items influence *L. lineolaris* performance (i.e., survivorship rate, developmental time, and adult weight and length). Improvements in fitness are indicated by higher nymphal survival, shorter developmental time, and larger adults. Immature stages of *L. lineolaris* show improved fitness when provided with diets rich in canola compared to strawberry flowers and spider mites. Furthermore, the inclusion of multiple diet items in phytozoophagous regimes enhances insect performance. The findings emphasize the significance of understanding *L. lineolaris’* nutritional requirements and the biodiversity of target ecosystems for modeling energy flows and designing effective IPM strategies against this pest. This research contributes to the knowledge base for biological control programs targeting *L. lineolaris* in agricultural systems.

## 1. Introduction

True omnivorous species exhibit a wide-ranging diet, consuming resources from different trophic levels, including both plants and animals [1]. Depending on their predominant feeding behavior, they can be classified as zoophytophagous when they primarily feed on animals or as phytozoophagous when they show a preference for plants [1]. Some omnivorous species possess the ability to adjust their feeding strategies based on food availability. Then, when resources are abundant, they forage for balanced macronutrients to maximize their fitness, while when resources are scarce, they present a reduced sensitivity to the constrains related to food availability [2]. As a result, true omnivores play a pivotal role in community and food web dynamics in natural and managed ecosystems [1,3].

Despite the widespread presence and significance of true omnivores, our current knowledge regarding the nutritional, functional, and temporal dynamics of complementary feeding and its implications for fitness remains incomplete [4,5,6]. In fact, a mixed diet consumed by true omnivores can be influenced by several factors [7,8]. Among them, environmental conditions such as resource availability, presence of competitors, predators, or multiple hosts are suspected to drive the food selection process [9,10]. However, biological factors, including sensorial preferences such as visual or olfactory [11], resource quality (e.g., plant nitrogen content) [12], toxin dilution [13], physiological and morphological adaptations (e.g., presence of digestive enzymes) [14,15], and intraspecific genetic variation [16], also influence diet choices. Furthermore, the behavior of omnivores can vary depending on the season, habitat, evolution from phytophagous or zoophagous species, life history stage, and individual condition [1,7,17].

In this context, the quality of host resources, including variations in quality between different host species and within the same species, is one of the main factors influencing dietary diversity, as it can significantly affect the performance of organisms [2,17]. Performance indicators include developmental time, survivorship rate, biomass accumulation, longevity, and fecundity of adults [18]. As demonstrated for several species, high-quality resources are expected to enhance individual survival, shorten development time, and increase longevity and reproduction [12,17]. 

Among mirid bugs (Hemiptera: Miridae), true omnivory is widespread and holds particular significance. This is because zoophytophagous mirids, such as those from the genera Nesidiocoris, Dicyphus, and Macrolophus, can function as beneficial natural enemies but may transition into pests under specific circumstances, whereas certain phytozoophagous mirids are recognized as significant agricultural pests [19,20,21,22,23,24]. This study focuses on the Nearctic pest *Lygus lineolaris* (Palisot de Beauvois) (Hemiptera: Miridae), which has gained economic importance due to its rapid population increase in North America. Factors such as high fecundity, short generation time, a wide host range, ability to survive under a broad range of temperatures, and developed resistance to insecticides contribute to its pest status [5,25,26,27,28]. *L. lineolaris* exhibits a polyphagous diet, feeding on up to 700 cultivated or non-cultivated plant species [28,29,30,31] and preying on several agricultural pests [4,26,32]. However, the limited understanding of *L. lineolaris* foraging behavior and the impact of their choices on their fitness, which in turn affects their distribution, population dynamics, and crop damage, presents significant challenges for the development and implementation of effective integrated pest management (IPM) strategies [4,8,33].

As an initial step towards understanding the dietary behavior of *L. lineolaris* and gaining insight into the complex nature of true omnivores, this study aimed to assess the nutritional quality of different diets and investigate the influence of supplemental food on the fitness of *L. lineolaris*. Furthermore, this study aimed to explore the potential utilization of *L. lineolaris’* nutritional requirements in the development of effective pest management strategies, particularly focusing on the incorporation of plant diversity with canola and buckwheat to mitigate the impact of this pest in strawberry fields. The specific subobjectives of the study included (1) evaluating the effects of different diets on survivorship rate, developmental time, body length, and weight of *L. lineolaris*, (2) investigating the impact of increasing diet diversity or augmenting the number of food items on performance, and (3) characterizing the predator behavior of *L. lineolaris*.

## 2. Materials and Methods

### 2.1. Diets

One flower of strawberry, (Fragaria × ananassa), canola (*Brassica napus*) and buckwheat (*Fagopyrum esculentum*) were offered as plant sources to *L. lineolaris*, while *Myzus persicae* (Sulzer) (Hemiptera: Aphididae) and *Tetranychus urticae* (Koch) (Prostigmata: Tetranychidae), provided ad libitum, were offered as animal hosts. Strawberry is an important crop in Canada and is highly affected by the three pests. Additionally, *L. lineolaris* preys on aphids and spider mites [32]. Canola and buckwheat bloom during the strawberry production season (May–September) and have shown to be effective lures to attract *L. lineolaris* [34,35,36]. 

### 2.2. Rearing

Tarnished plant bug (TPB), *L. lineolaris,* breeding was initiated with individuals captured from field margins in southeastern Quebec (Canada) at the beginning of the summer season. In the laboratory, *L. lineolaris* was reared on organic romaine salad, and individuals were separated into different age ranges (L1–L3, L4–L5, adults < 7 days, and adults > 7 days) in small ventilated containers (6″ × 6′ × 4″) under controlled conditions. Spider mites, *T. urticae*, were purchased from Anatis Bioprotection (Saint-Jacques-le-Mineur, Quebec, Canada) and directly used in the assays, while green peach aphids, *M. persicae*, were raised in the laboratory on eggplant plants. 

Plants were grown in greenhouses from seeds (for canola and buckwheat) and cuttings (for strawberry) in a mixture of compost and soil and watered daily. Rearing procedures and experiments were conducted under standard environmental conditions in a growth cabinet at 25 °C, 70% RH, and 16:8 h (L:D) photoperiod.

### 2.3. Biological Trials

Experiments were conducted under standard environmental conditions in a growth cabinet at 25 °C, 70% RH, and 16:8 h (L:D) photoperiod. Daily, newly emerged *L. lineolaris* L1 nymphs were isolated in small containers with romaine salad. After five days, L2 nymphs were individually transferred to test arenas. The test arenas consisted of aerated petri dishes (9 mm diameter) containing agar gel to maintain moisture, a strawberry leaf with the abaxial side exposed, which served as substrate, a specific diet (treatment), and a moistened cotton pad as water source. Nine different treatments, including plant, animal, or mixed diets, were tested (Table 1). Each treatment consisted of 20 individuals, except for the “canola + aphids” treatment, which had 19 individuals. Each individual was treated as a separate replicate. The nymphs were allowed to feed ad libitum until they reached the adult stage or died, with daily recording of their progress. Plant and mixed diet treatments exclusively included the flower structure. Twenty aphids and/or spider mites were offered in the zoophagous or phytozoophagous diets to ensure an ad libitum nutrient source [32]. To ensure optimal conditions of diets (i.e., flower freshness and/or ad libitum preys), individuals were transferred to new petri dishes with fresh agar and diet every 2–3 days. 

Upon adult emergence, individual measurements of weight, sex, and body length were taken. These insects were briefly exposed to CO_2_ for ease of manipulation. Weight was measured using a precision balance, and photographs were taken under a binocular microscope for later evaluation of sex and body length using Image J [37]. Three measurements of body length and weight were taken per insect, and the averages of these values were used for analysis.

Several variables were measured to assess *L. lineolaris*’ fitness on different diets, including duration of nymphal development from the N2 stage to adulthood, nymphal survival rate, nymphal survival curves, sex ratio, adult weight, and adult body length. 

### 2.4. Statistical Analyses

**Figure 1:**
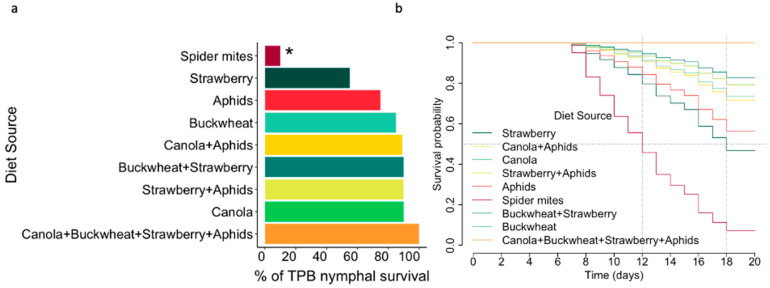
*L. lineolaris* nymphal survival rates and survival curves according to diet sources. (**a**) Percentage of survivorship of *L. lineolaris* nymphs. Asterisk (*) denote statistical differences among diets (Tukey, alpha = 0.05). (**b**) Survival probability curves of *L. lineolaris* nymphs over time (days). Median survival times are indicated by dotted lines.

Diets were categorized into three feeding regimes (Phyto-Zoophagous, Zoophagous, and Phytophagous) and three categories of food items, regardless of the diet source (one, two, or four diet items) (Table 1). Spider mites were excluded from the analysis of feeding regimes and food items due to their significant negative impact on *L. lineolaris* survivorship, with only two individuals surviving to adulthood. (Figure 1). Nymph survivorship was analyzed using a generalized linear model with a binomial distribution for the variable ‘number of dead nymphs out of the total number of nymphs studied’ per diet. In these models, the treatment (i.e., diet, feeding regime, or food items) was the dependent variable. Multiple comparisons with Tukey’s test were used to assess differences among treatments. Kaplan–Meier survival curves, along with the Cox proportional hazards model [38], were utilized to evaluate the influence of diet, feeding regime, and food items on nymphal survivorship. Censored data points were included for individuals reaching adulthood without death. 

For the adult measurements, Kruskal–Wallis tests were applied to explore developmental time to adulthood, sex, weight, and body length. In these models, the treatment variable (diet, feeding regime, or food items) served as the dependent variable. Due to insignificant differences in developmental time, body weight, and length between sexes on different diets (X^2^_1_ = 1.922, *p* = 0.166, X^2^_1_ = 1.132, *p* = 0.287, X^2^_1_ = 1.714, *p* = 0.287, respectively), males and females were combined. Means were compared using a Bonferroni-corrected Wilcoxon test. Furthermore, a Spearman correlation analysis was conducted to evaluate the relationships between the number of days to adulthood and adult measures (weight and body length). For all models, nymphs fed on strawberries, phytophagous feeding regime, or a single diet item were used as the reference treatment. The models underwent verification and adjustment based on diagnostic plots, including residual plots and histograms. Proportional hazards assumption was confirmed by analyzing Schoenfeld residuals and plotting scaled residuals against time. 

Principal component analysis (PCA) was performed on the number of days to adulthood, weight, and body length to classify *L. lineolaris* specimens based on their fitness condition and to identify the primary dietary factors (i.e., different diets, feeding regimes, and the number of diet items) influencing their developmental outcomes, thus reducing noise and clarifying the overall trends. The multivariate data were transformed with Tukey’s Ladder of Powers transformation. Permanova analysis with Bray Curtis distance was used to assess the significance of multivariate effect on diet, regime, and number of items. 

All statistical analyses were performed using R software (version 4.1.3) with various libraries including emmeans, lme4, car, factoextra, FactorMineR, Hmisc, MuMIn, MASS, rcompanion, survminer, survival, vegan, and ggplot2. The significance level for all tests was set at alpha = 0.05.

## 3. Results

### 3.1. Diet Source 

Feeding on spider mites resulted in a significant decrease in *L. lineolaris* nymphal survival (log odds: −2.39, Z = 2.755, *p* < 0.01). In contrast, the other diets were associated with a significant increase in survival (*p* < 0.05), except for the ‘canola + buckwheat + strawberry + aphids’ and ‘aphids’ diets, where no significant differences were observed compared to the other treatments (Table 2 and Figure 1a). When comparing to feeding on strawberry flowers alone, feeding on spider mites significantly decreased the probability of nymph survival (hazard ratio (HR): 3.44 ± 0.4, *p* < 0.005). However, other diets showed an increasing trend in nymph survival, although no significant differences with strawberry were found (Table 2 and Figure 1b). Notably, the diet mixture “canola + buckwheat + strawberries + aphids” exhibited the highest probability of survival (lowest HR) compared to feeding on strawberries alone. The median survival times for *L. lineolaris* nymphs were 12 days when fed on spider mites and 18 days when fed on strawberries, while individuals fed on the rest of the diets had survival times exceeding 20 days (Figure 1b).

**Figure 2:**
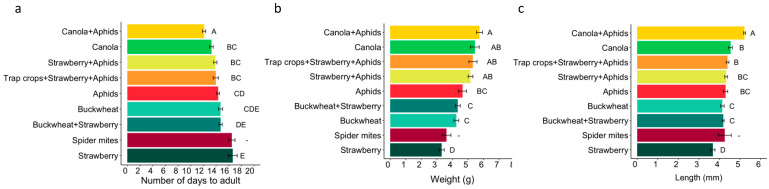
Number of days to adulthood (**a**), adult body weight (g), (**b**) and adult body length (mm) (**c**), according to diet with error bars (±SE). Different letters denote statistical differences in Wilcoxson test with Bonferroni adjustment (alpha = 0.05). Note that “Trap Crops” stands for “Canola+Buckwheat”.

Spearman correlation analysis revealed an inverse relationship between the number of days to adulthood and adult body measures (i.e., body weight and length) (r^2^ = −0.42, *p* < 0.0001 for adult weight; r^2^ = −0.54, *p* < 0.005 for body length), while the two adult measures were positively correlated (r^2^ = 0.64, *p* < 0.0001). A 37.3% difference in developmental time to adulthood among diets was observed. The shortest developmental time to adulthood was observed in *L. lineolaris* fed on the phytozoophagous mixture ‘canola + aphids’ (12.12 ± 0.27 days), while the longest time (~16 days) was recorded for *L. lineolaris* fed on strawberry flowers or spider mites (Table 2, Figure 2a). The increase in plant diversity in a diet did not significantly reduce the developmental time to adulthood. Yet, no significant differences were observed among the mixtures ‘buckwheat + strawberry’ or ‘canola + buckwheat + strawberry + aphids’ compared to ‘buckwheat’ or ‘strawberry + aphids’. Furthermore, the three phytophagous diets tested presented significant differences in developmental time to adulthood: *L. lineolaris* individuals fed with canola had the shortest developmental time, while those fed with strawberry had the longest, and those fed on buckwheat had an intermediate developmental time. Additionally, a noticeable 42% difference in body weight was observed among diets. The heaviest *L. lineolaris* adults were those fed on phytozoophagous diets (phytozoophagous average weight = 5.32 g) and canola (5.44 ± 0.28 g), while the lightest adults were those fed on strawberry (3.31 ± 0.17 g) and spider mites (3.62 ± 0.28 g). Adults fed with diets containing buckwheat (4.25 ± 0.16 g) or only aphids (4.65 ± 0.25 g) had intermediate weights (Table 2, Figure 2b). A 29.5% difference in body lengths was observed among the diets. The diets containing canola (mixed or alone) resulted in the largest individuals (5.22 ± 0.05 mm, 4.53 ± 0.1 mm, respectively), slightly larger than those fed on aphids (4.30 ± 0.1 mm) or buckwheat (4.14 ± 0.09 cm). The strawberry diet provided the shortest adults (3.68 ± 0.12 mm) (Table 2, Figure 2c).

The sex ratio did not differ significantly among treatments (X^2^_8_ = 14.58, *p* = 0.068). Out of the 136 *L. lineolaris* individuals that completed development to the adult stage, 61 were females and 75 were males, indicating an approximate sex ratio close to 1:1. A tendency, not statistically supported, where females are heavier and larger than males was observed. 

### 3.2. Feeding Regimes

**Figure 3:**
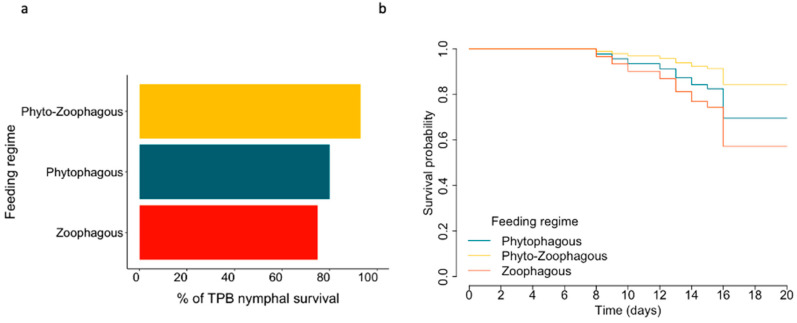
*L. lineolaris* nymphal survival rates and survival curves according to feeding regimes. (**a**) Percentage of survival of *L. lineolaris* nymphs. (**b**) Survival probability curves of *L. lineolaris* nymphs over time (days). Median survival times exceeded 20 days.

The feeding regime influenced nymphal survival (Table 2). However, no significant differences were found between specific groups or treatments upon further investigation using Tukey’s test (Figure 3a). A trend was observed where the phytozoophagous regime increased nymphal survival, while the zoophagous regime decreased survival compared to the phytophagous regime (log odds: 1.24, Z = 2.10, *p* = 0.08 and log odds = −0.29, Z = −0.49, *p* = 0.97, respectively).

**Figure 4:**
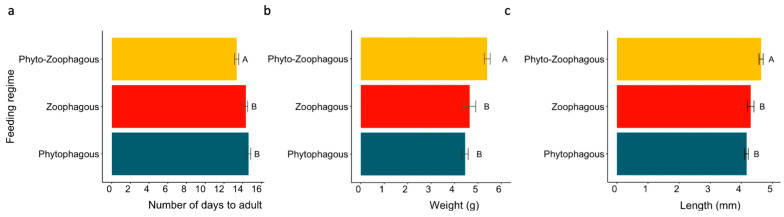
Number of days to adulthood (**a**), adult body weight (g) (**b**), and adult body length (mm) (**c**), according to feeding regimes with error bars (±SE). Different letters denote statistical differences in Wilcoxson test with Bonferroni adjustment (alpha = 0.05).

Similarly, the survival curves of *L. lineolaris* nymphs were not significantly affected by the feeding regimes (Table 2). However, when compared to the phytophagous regime, the phytozoophagous feeding regime increased the probability of nymph survival by a hazard ratio (HR) of 0.46. In contrast, the zoophagous feeding regime decreased the probability of nymph survival by an HR of 1.54 (Figure 3b). Overall, a phytozoophagus feeding regime significantly decreased the number of days required to become adult and significantly increased adult body weight and length compared to single feeding regimes, regardless of whether they were zoophagous or phytophagous (Table 2 and Figure 4). 

### 3.3. Number of Diet Items

**Figure 5:**
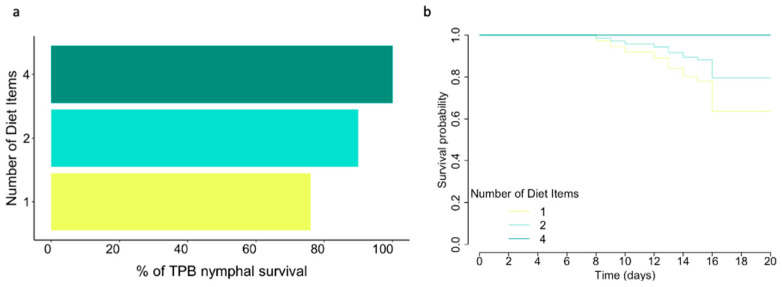
*L. lineolaris* nymphal survival rates and survival curves according to number of diet items provided. (**a**) Percentage of survival of *L. lineolaris* nymphs. (**b**) Survival probability curves of *L. lineolaris* over time (days). Median survival times exceeded 20 days.

The survival rate increased with the number of hosts (Table 2), exhibiting a statistically significant increase in log odds of 1.01 when feeding on two substrates (*p* < 0.05) and a notable increase of 24.59 when feeding on four substrates (not statistically significant). However, upon conducting multiple comparisons using Tukey’s test, no significant differences were found between specific groups or treatments (Figure 5a). Feeding on more than a single item increased the probability of nymphal survival (HR < 0.5) (Table 2 and Figure 5b). A median survival time exceeding 20 days was observed regardless of the number of diet items offered.

**Figure 6:**
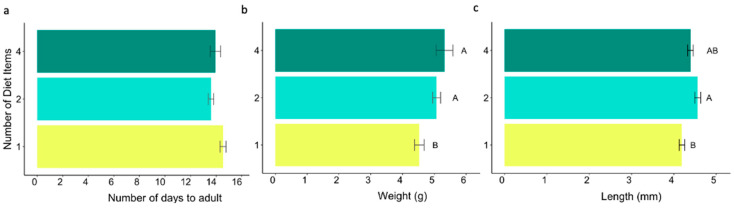
Number of days to adulthood (**a**), adult body weight (g) (**b**), and adult body length (mm) (**c**), according to feeding regimes with error bars (±SE). Different letters denote statistical differences in Wilcoxson test with Bonferroni adjustment (alpha = 0.05).

A tendency where feeding on more than a single diet decreased the number of days to adulthood and significantly increased adult body weight and length was observed (Table 2 and Figure 6).

### 3.4. PCA Analysis

**Figure 7:**
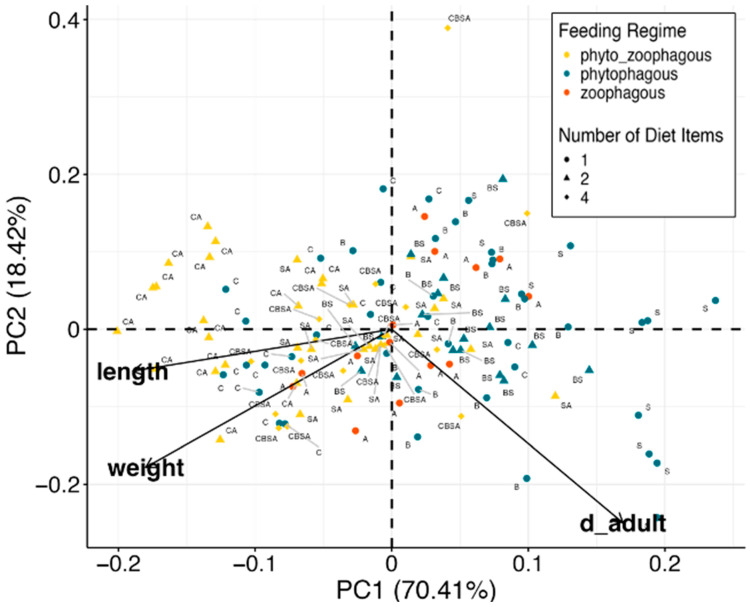
PCA of feeding regimes, number of diet items, and diet sources tested. Different diets are represented with the following letters: (i) CBSA: Canola + Buckwheat + Strawberry + Aphid, (ii) SA: Strawberry + Aphid, (iii) C: Canola, (iv) B: Buckwheat, (v) S: strawberry, (vi) BS: Buckwheat + Strawberry, (vii) A: Aphid, (viii) CA: Canola + Aphid. Feeding regimes are represented by different colors while number of diet items by different shapes. The direction of the tested factors, number of days to adult (d_adult) and *L. lineolaris* adult allometric measures (body weight and body length), are represented. Spider mites are not included in the analysis.

The PCA analysis highlighted the impact of different feeding regimes by organizing the individuals along principal components, thus reducing noise and clarifying the overall trends. This analysis revealed that the first principal component, which explained 70.41% of the total variance, represents *L. lineolaris* fitness (Figure 7). Individuals on the right side of this first component (x-axis) had longer developmental times and smaller, lighter bodies, whereas those on the left side were larger, heavier, and had shorter developmental times.

The results showed that a phytozoophagous feeding regime provided the best fitness for *L. lineolaris*, indicated by heavier, longer insects with shorter developmental times, especially when canola was part of the diet. Furthermore, PCA showed that mixing several diets (CBSA) resulted in lower fitness compared to simpler diets with only two components (CA or SA) (Table 1 and Figure 7). Conversely, the worst fitness was observed with a diet consisting only of strawberry (S). This low fitness was slightly improved with the addition of buckwheat (BS) and significantly enhanced with the addition of aphids (SA). However, mixing buckwheat and strawberry (BS) did not result in an increase in fitness compared to feeding on buckwheat alone (B) (Figure 7). 

## 4. Discussion

In this study, we focused on the inherent nutritional quality of diet sources and their impact on the fitness of *L. lineolaris* by conducting experiments in a controlled laboratory setting. Our results generally showed that mixing diets offered an improvement in the fitness of *L. lineolaris* compared to single diets, particularly when the mix included both zoophagous and phytophagous feeding regimens. This improvement was evident with increased nymphal survival, shorter developmental time, and larger adult size compared to feeding solely on a plant or animal-based diet. These findings not only align with previous studies on other species [12,39] but also support the nutrient-balanced diet hypothesis, which suggests that mixing diets promotes consumer fitness by complementing nutrients and diluting toxic compounds [6]. 

Miridae are one of the major families of omnivorous insects [40]. Unlike other omnivorous Miridae (Hemiptera: Miridae), like *Campylomma verbasci* (Meyer-Dür) or *Dicyphus hesperus* (Knight), which require animal sources to complete their development [21,41], our study found that *L. lineolaris* can successfully reach adulthood by consuming both plant and animal food sources. Plant and animal consumption were not directly assessed in this study. However, Solà et al. [32] found that *L. lineolaris* preyed on spider mites and aphids, consuming up to 35 individuals when given 50 aphids and 20 prey when offered 40 spider mites. In the present study, plant consumption was inferred from the fact that most *L. lineolaris* individuals reached adulthood without alternative sources of nutrition. No significant differences were found in fitness parameters between plant-based and animal-based feeding regimes for *L. lineolaris*, indicating that phytophagy or zoophagy during its immature stages is more of an opportunistic behavior than a necessity. Then, *L. lineolaris,* as do several omnivores, may exhibit reduced sensitivity to food availability constraints [2,33]. This reduced sensitivity can impact the species’ resilience and potentially lead to an increase in pest-related issues. Nevertheless, classifying *L. lineolaris* as either phytozoophagous or zoophytophagous based solely on diet quality is arbitrary, despite its status as an important pest species. 

Some studies have supported the nutrient-balanced diet hypothesis only when prey is added to poor-quality plant diets [42]. However, our study revealed that combining high-quality (canola) and low-quality (strawberry) plant resources with aphids increased the fitness of *L. lineolaris* and exceeded the outcomes of relying solely on any one of the three food sources. Moreover, Lindquist and Sorensen [43] demonstrated that the presence of aphids on plants enhances their attractiveness to TPB. Predators utilize their prey not just for energy but also as a means to acquire essential nutrients for somatic growth and development [2]. Interestingly, when strawberry was added to buckwheat, there was no significant difference in fitness compared to a diet consisting exclusively of buckwheat. These findings highlight the importance of food diversity in enhancing the fitness of *L. lineolaris*, surpassing the significance of the number of available food items. Furthermore, the nutritional composition of different food sources, whether plant or animal, contributes to this impact. 

Due to its status as a pest, previous research on the contribution of predation to the diet of *L. lineolaris* has been limited [44]. Our findings confirm that a phytophagous diet complemented with *M. persicae* enhances TPB fitness. A similar result has been reported for *L. rugulipennis* [45]. Nevertheless, while *L. lineolaris* can resort to feeding on *T. urticae* when no other resources are available [32], the present study showed that it rarely reaches adulthood when relying solely on this food source. Therefore, similar to findings for other species, *T. urticae* should be considered harmful to *L. lineolaris* immatures [41], and diversifying *L. lineolaris’* diet should solve this problem. 

Importantly, the quality of the diet may not remain consistent. Yet, food quality can fluctuate over time, influenced by factors such as temperature, plant phenology, or seasonal availability [9,46,47]. Furthermore, food quality can be also affected by consumer’s age [41]. For instance, Oberhauser et al. [46] found that the levels of defensive compounds of milkweed, monarch butterflies’ *Danaus plexippus* Linnaeus (Lepidopera: Nymphalidae) primary food source, changes throughout the growing season, affecting the growth and survival of monarch caterpillars. Aubry et al. [41] found that *C. verbasci* feeding on mixed diets containing living aphids resulted in high insect mortality in the first nymphal instar, likely due to the young stages’ inability to handle aphids despite their nutritional value. Additionally, some plants produce toxic compounds, such as alkaloids or phenolics, as a defense mechanism against herbivory [48]. In instances where prey population exponentially increases in dietary mixtures, the plant resource could trigger the release of such defensive compounds, compromising the overall diet quality and consequently deterring herbivory. This phenomenon has been observed when *Frankliniella occidentalis* (Pergande) (Thysanoptera: Thripidae) reduced feeding on cotton leaves in the presence of spider mites due to the induction of toxic plant defenses caused by spider mite feeding on the cotton plant [49]. Consequently, predicting the movement of omnivorous species like *L. lineolaris* to alternative host resources for pest management purposes can be a complex task. 

Landscape significantly influences the movement and dispersal of insects within agroecosystems. As a result, strategic landscape management can be effectively employed as an integrated pest management strategy to reduce the presence of pests on crops [50]. This is particularly evident in the Mississippi River Delta region, where 169 plant species from 35 families, primarily consisting of herbaceous non-crop plants, have been identified as hosts for *L. lineolaris* [51]. Moreover, Dumont and Provost [35,52] found that *L. lineolaris* is more abundant on buckwheat, mullein, and mustard compared to strawberries. With this in mind, the present study offered canola, buckwheat and strawberries as phytophagous diet sources to assess the nutritional quality of these options for *L. lineolaris* and to determine whether its dietary choices were driven by an improvement in fitness, as predicted by optimal foraging theory. Results revealed that canola and buckwheat, particularly canola, improved the fitness of *L. lineolaris* compared to strawberries. Thus, *L. lineolaris* dietary choices are driven, though not exclusively, by an improvement in fitness. Additionally, the use of canola and buckwheat as trap crops to divert *L. lineolaris* away from economical crops should be further studied. Firstly, canola and other highly nutritious plants attract several insect species by enhancing their fitness, which can trigger population outbreaks regardless of their ecological role (i.e., pest or natural enemy). These populations can be locally reduced using insecticides or vacuuming [35,52,53]. On the other hand, naturally occurring or introduced natural enemies, employed as a biological control strategy, could help reduce *L. lineolaris* populations. However, in such situations, to avoid predation risk, *L. lineolaris* may move to less profitable hosts, such as strawberries, which can lead to crop damage. Secondly, these plants also attract potential prey for *L. lineolaris*, such as aphids or spider mites. While these preys can increase host attractiveness and improve the fitness of *L. lineolaris*, they can also activate plant defense signaling pathways, deterring *L. lineolaris* [48] and diverting the individuals to other plants. Additionally, the presence of alternative prey can increase competition, potentially resulting in intraguild predation and further affecting the redistribution of *L. lineolaris* in the field [54].

Overall, this study underscores the significant influence of diet sources on the fitness of the agricultural pest *L. lineolaris*. Our findings show that canola-rich diets enhance survivorship, reduce developmental time, and increase adult length and weight, while feeding exclusively on strawberries results in poor insect fitness. Understanding specific nutritional requirements and their relationship to fitness parameters is crucial for developing effective integrated pest management strategies against omnivorous species such as *L. lineolaris*.

## Figures and Tables

**Table 1:** Feeding regime, diet source, number of diet items offered to *L. lineolaris*, and treatment sample size. Numbers in square brackets denote sample size, while letters in brackets indicate diet components abbreviations.

Feeding Regime	Diet Source [20 *]	Number of Diet Items
Phyto-Zoophagous [59]	Canola + Buckwheat + Aphids + Strawberry (CBSA)	4 [20]
	Canola + Aphids (CA)	2 [59]
	Strawberry + Aphids (SA)	
Phytophagous [80]	Buckwheat + Strawberry (BS)	
	Canola (C)	1 [80]
	Buckwheat (B)	
	Strawberry (S)	
Zoophagous [20]	Aphids (A)	
	Spider mites ** (M)	

* Except for (CA) [19]. ** Spider mites were not included in the feeding regime or the number of diets fed analyses, as only 2 individuals survived to adulthood.

**Table 2:** Model results of the analyses for various fitness measures based on diet sources, feeding regimes (phytozoophagous, phytophagous, or zoophagous), and the number of diet items offered (one, two, or four). For nymph survivorship, X^2^ represents the deviance from the generalized linear model (GLM) for binomial data. For nymph developmental time, adult body weight, and adult body length, X^2^ indicates the chi-squared statistic from the Kruskal–Wallis test. LR denotes likelihood ratio test from the Cox proportional hazards model.

Fitness Measure	Diet (df = 8)		Feeding Regime (df = 2)		Number of Diet Items (df = 2)	
	Statistic	*p*-Value	Statistic	*p*-Value	Statistic	*p*-Value
Nymph survivorship	X^2^ = 65.65	***	X^2^ = 6.54	*	X^2^ = 11.84	*
Nymph survival curves	LR = 46.14	***	LR = 3.51	0.2	LR = 9.05	*
Nymph development time	X^2^ = 57.40	**	X^2^ = 17.22	**	X^2^ = 5.70	0.06
Adult body weight	X^2^ = 55.70	**	X^2^ = 24.73	***	X^2^ = 11.01	*
Adult body length	X^2^ = 69.94	**	X^2^ = 23.84	**	X^2^ = 12.00	*

*** < 0.0001, ** < 0.001, * < 0.05.

## Data Availability

The raw data presented in this study are available upon request from the corresponding author.
